# Heart rate variability as an indicator of COVID-19 induced myocardial injury: a retrospective cohort study

**DOI:** 10.1186/s12871-023-01975-8

**Published:** 2023-01-10

**Authors:** Hani Taman, Nabil Mageed, Mohamed Elmorsy, Sherif Elfayoumy, Mostafa Elawady, Ahmed Farid, Mohamed Abdelmonem, Ibrahim Abdelbaser

**Affiliations:** 1 Department of Anesthesia and Surgical Intensive Care, Faculty of Medicine, Mansoura University, 2 El-Gomhouria Street, Mansoura, 35516 Egypt; 2 Department of Anesthesia and Surgical Intensive Care, Faculty of Medicine, Damietta University, Damietta, Egypt; 3 Department of Anesthesia and Surgical Intensive Care, Faculty of Medicine, Portsaid University, Portsaid, Egypt; 4 Swansea University Medical School, Swansea, UK

**Keywords:** Autonomic nervous system, COVID-19, Heart rate, Intensive care units, Length of stay

## Abstract

**Background:**

Heart rate variability (HRV) is a valuable indicator of autonomic nervous system integrity and can be a prognostic tool of COVID-19 induced myocardial affection. This study aimed to compare HRV indices between patients who developed myocardial injury and those without myocardial injury in COVID-19 patients who were admitted to intensive care unit (ICU).

**Methods:**

In this retrospective study, the data from 238 COVID-19 adult patients who were admitted to ICU from April 2020 to June 2021 were collected. The patients were assigned to myocardial injury and non-myocardial injury groups. The main collected data were R-R intervals, standard deviation of NN intervals (SDANN) and the root mean square of successive differences between normal heartbeats (RMSSD) that were measured daily during the first five days of ICU admission.

**Results:**

The R-R intervals, the SDANN and the RMSSD were significantly shorter in the myocardial injury group than the non-myocardial group at the first, t second, third, fourth and the fifth days of ICU admission. There were no significant differences between the myocardial injury and the non-myocardial injury groups with regard the number of patients who needed mechanical ventilation, ICU length of stay and the number of ICU deaths.

**Conclusions:**

From the results of this retrospective study, we concluded that the indices of HRV were greatly affected in COVID-19 patients who developed myocardial injury.

## Background

Coronavirus (COVID-19) outbreaks result in thousands of deaths worldwide [1, 2]. Although few patients remain asymptomatic, others may develop non-respiratory symptoms resulting from hepatic, renal, or cardiac dysfunction [3].

To get an intracellular access, COVID-19 viral surface spike protein binds to the human angiotensin-converting enzyme 2 (ACE2) receptor which are highly expressed in the heart [4]. ACE2 role is to counteracts angiotensin II effects in excessive renin-angiotensin system activation in certain circumstances as hypertension (HTN) and congestive heart failure (CHF) [5]. Additionally, COVID-19 can cause impairments to the cardiovascular system because of autonomic nervous system dysfunction. This is presented as acute myocardial infarction, myocarditis and thromboembolic events. COVID-19 induced myocardial injury, can be diagnosed by elevated cardiac biomarkers, new ECG changes, or abnormal echocardiographic findings.

Heart rate (HR) is a dynamic signal that depends on the autonomic nervous system, which modulates the cardiac activity [6]. A high range of variability in HR indicates good functioning autonomic nervous system, while a low HRV means an abnormal autonomic system in various of medical conditions, including ischemic heart disease (IHD), myocarditis and congestive heart failure [7, 8]. The degree and severity of the changes in HRV may be correlated with the severity of the illness and can provide a valuable prognostic indicator of infection in the critically ill patients [9]. The evaluation of cardiac autonomic function integrity and the degree of heart rate variation can help the early identification of myocardial affection in seriously ill patients with heavy viral infections [10].

This retrospective study was performed on the data derived from COVID-19 patients who were admitted to intensive care unit (ICU) and compared HRV indices between patients who developed myocardial injury and those without myocardial injury.

## Methods

### Study design and population

This retrospective observational cohort study was conducted at Mansoura University hospital, intensive care unit after approval from the Mansoura Faculty of Medicine Institutional Research Board (code number R.21.05.1332) on July 27, 2021, to collect and publish the data concerning this study. We included in this the data from all COVID-19 adult patients who were admitted to intensive care unit (ICU) from April 2020 to June 2021 (238 patients). We excluded all patients with diseases that may affect HRV, including known cardiac diseases (IHD, CHF, myocarditis or arrhythmias) within last six months prior COVID-19 infections, and diabetes mellitus. The need for informed consent was waved by the local institutional review board.

The infection with COVID-19 virus was confirmed by the nasopharyngeal swabs using the real-time reverse-transcriptase polymerase chain reaction assays. The ICU admission criteria were, the need for mechanical ventilation or non-invasive ventilation for more than 2 h, respiratory distress (tachypnea or the need for oxygen > 6 L/min to maintain SpO2 > 92 or PaO2 > 65), hemodynamic instability requiring vasopressor support, impaired level of consciousness, acidosis either metabolic or respiratory, more than organ failure and patients with abnormal ECG findings, including ischemia and arrhythmia. The data from eligible patients were classified into 2 groups:

A- The myocardial injury group: patients had at least 2 of the following criteria, elevated cardiac enzymes, including creatine kinase > 160 U/L, CK-MB isoenzyme > 5.0 ug/L and troponin level > 0.098 ng/ml), ST elevation more than 2 mm and transthoracic echocardiographic study (left ventricular systolic dysfunction with reduced ejection fraction (< 35%), wall motion abnormalities and diastolic dysfunction).
B- Non-myocardial injury group: patients did not have myocardial injury.

### Analysis of HRV

Dynamic Electrocardiography (DCG) criteria were used to diagnose HRV on ECG sheets of all patients. The time-domain indicators of HRV were used for analysis: a) The time domain analysis included: mean of R-R intervals for normal beats (0.6–1.2 s; b) SDNN (standard deviation of NN intervals), the standard deviation of consecutive regular R-R intervals in 5 min (127 ± 35 ms) [11]. c) RMSSD, the root mean square of successive heart beat interval differences in 24 h ( 30 ± 12 ms) [12, 13].

### Data collection

The primary outcome measures were the indices of heart rate variability, including the mean of R-R intervals, SDNN and RMSSD that were measured daily during the first five days of ICU admission. The secondary outcome measures were the criteria of myocardial injury (serum level of CK, CKMB and troponin, EF and ST segment elevation) that were determined during the fifth day of ICU admission, the number of patients who needed mechanical ventilation, ICU length of stay and the number of ICU deaths.

### Data management and analysis

Statistical analysis performed using IBM SPSS for Windows, Version 22.0 (IBM Corp., Armonk, NY, USA). Data normality was tested using Kolmogorov–Smirnov test. Chi-square or Fisher’s exact test were used for categorical data analysis. Continuous normally distributed data were analyzed using independent sample t-test. Pearson’s correlation was used to assess the correlation between variables. Continuous variables were summarized using mean ± standard deviation (SD). Categorical variables were expressed as frequencies and percentage. *P* < 0.05 was considered statistically significant.

## Results

**Fig. 1:**
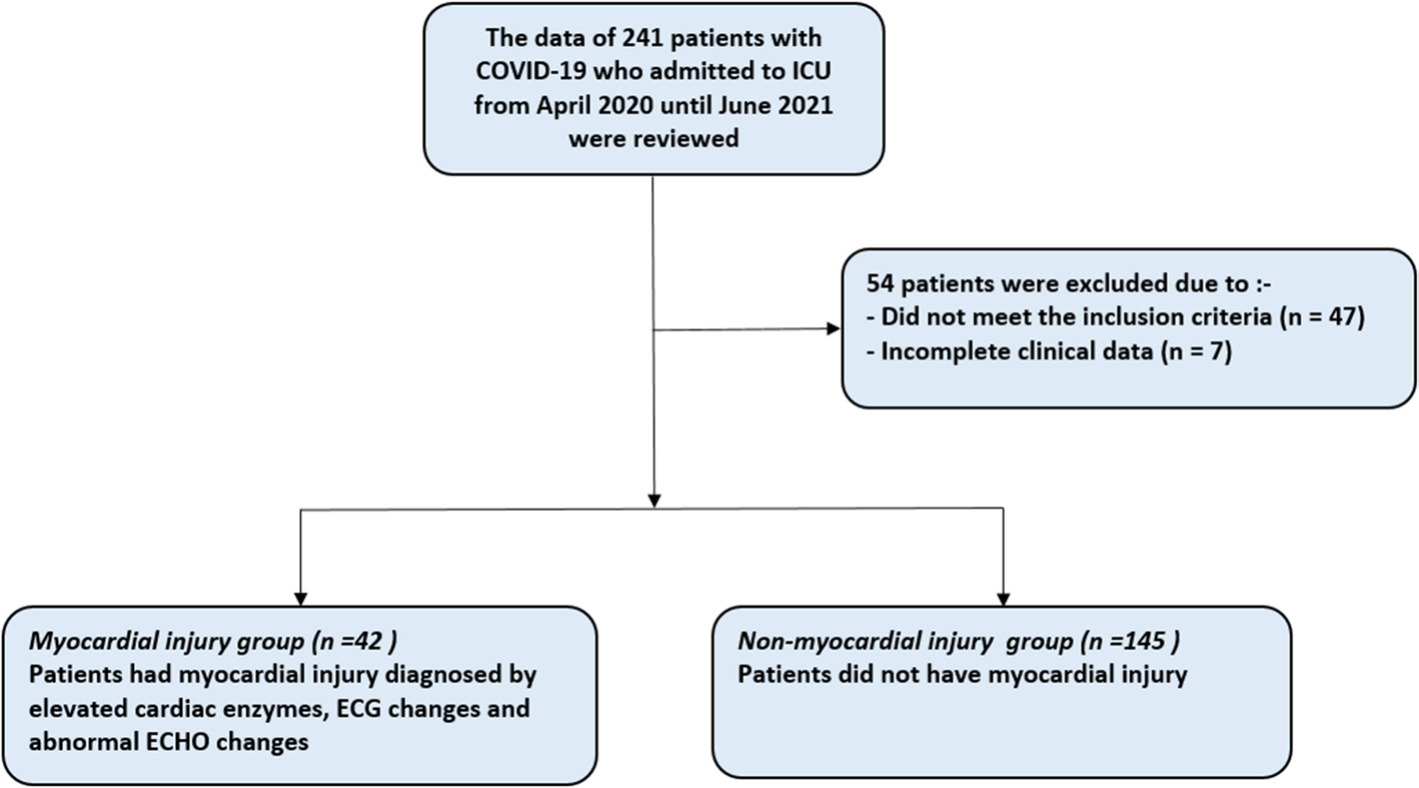
Study Flowchart

The data from 241 COVID-19 patients who were admitted to ICU were retrospectively reviewed, of whom 54 were excluded either due to the absence of the fulfilling inclusion criteria (*n* = 47) or the presence of incomplete data (*n* = 7) (Fig. 1). The final analysis was done on the data from 187 patients, myocardial injury group (*n* = 42) and non-myocardial injury group (*n* = 145) (Fig. 1).

There were no significant differences between the myocardial injury and the non-myocardial injury groups in terms of patients’ characteristics, including age, gender, and the number of ICU deaths (Table 1).

**Table 1:** Patients characteristics, Co-morbidity, Mechanical ventilation, ICU length of stay, ICU death of the studied groups

	Myocardial injury group (*n* = 42)	Non-myocardial injury group (*n* = 145)	*P* value
Age, year	42.29 ± 6.97	44.26 ± 7.83	0.145
Gender M/F, n	27/15	92/53	0.064
*Criteria of myocardial injury*			
CK, U/L	603.34 ± 157.98	214.47 ± 15.67	0.001*
CKMB, ng/ml	22.58 ± 5.45	7.72 ± 1.71	0.002*
Troponin level, ng/ml	66.34 ± 21.29	22.53 ± 7.29	0.004*
EF, %	34.09 ± 9.08	43.38 ± 4.69	0.001*
ST elevation (mm)	3.88 ± 1.19	1.98 ± 0.65	0.002*
ICU deaths, n (%)	10/42 (23.8)	12/145 (8.2)	0.051

Data are expressed as mean ± SD and number (n) and percentage (%)

*CK* Creatine kinase, *CKMB* Creatine Kinase-MB, *EF* Ejection Fraction, *ICU* Intensive Care Unit

^*^ *p* < 0.05 is statistically significant

The criteria of the myocardial injury are shown in Table 1. The mean (SD) serum levels of CK (U/L), CKMB (ng/ml) and troponin (ng/ml) were significantly higher (*p* < 0.05) in the myocardial injury group than the non-myocardial group (603.34 ± 157.98 vs 214.47 ± 15.67, 22.58 ± 5.45 vs 7.72 ± 1.71 and 66.34 ± 21.29 vs 22.53 ± 7.29 respectively). The mean (SD) EF (%) was significantly lower (*p* = 0.001) in the myocardial injury group (34.09 ± 9.08) than the non-myocardial injury group (43.38 ± 4.69). The mean (SD) ST segment elevation (mm) was significantly higher (*p* = 0.002) in the myocardial injury group (3.88 ± 1.19) than the non-myocardial injury group (1.98 ± 0.65) (Table 1).

The mean (SD) R-R intervals (ms) measured at the first, second, third, fourth, and the fifth days of ICU admission were significantly shorter (*p* < 0.05) in the myocardial injury group than the non-myocardial injury group (498.09 ± 42.28 vs 556.01 ± 29.84, 535.65 ± 43.00 vs 618.61 ± 52.16, 584.96 ± 45.32 vs 634.96 ± 42.86, 522.38 ± 43.64 vs 609.29 ± 36.38 and 653.41 ± 28.33 vs 565.68 ± 53.17 respectively) (Table 2).

**Table 2:** The R-R intervals, the SDNN and the RMSSD of the studied group

	Myocardial injury group (*n* = 42)	Non-myocardial injury group (*n* = 145)	*P* value
*First day*			
R-R intervals (ms)	498.09 ± 42.28	556.01 ± 29.84	0.001*
SDNN (ms)	64.17 ± 10.91	101.36 ± 23.91	0.003*
RMSSD (ms)	17.27 ± 2.83	26.68 ± 5.33	0.003*
*Second day*			
R-R intervals (ms)	535.65 ± 43.00	618.61 ± 52.16	0.004*
SDNN (ms)	67.07 ± 12.78	100.07 ± 26.11	0.001*
RMSSD (ms)	18.05 ± 2.89	28.36 ± 5.42	0.002*
*Third day*			
R-R intervals (ms)	584.96 ± 45.32	634.96 ± 42.86	0.003*
SDNN (ms)	70.63 ± 13.77	106.84 ± 28.00	0.004*
RMSSD (ms)	19.51 ± 3.36	27.19 ± 5.63	0.006*
*Fourth day*			
R-R intervals (ms)	522.38 ± 43.64	609.29 ± 36.38	0.007*
SDNN (ms)	74.94 ± 13.99	100.49 ± 19.99	0.009*
RMSSD (ms)	18.41 ± 2.69	26.95 ± 5.39	0.001*
*Fifth day*			
R-R intervals (ms)	653.41 ± 28.33	565.68 ± 53.17	0.153
SDNN (ms)	69.87 ± 12.23	92.80 ± 23.21	0.008*
RMSSD (ms)	20.62 ± 4.04	28.12 ± 5.38	0.008*

Data are expressed as mean ± SD

*SDNN* Standard Deviation of NN intervals, *RMSSD* Root Mean Square of the Successive Differences

^*^ *p* < 0.05 is statistically significant

The mean (SD) SDNN (ms) measured at the first, second, third, fourth, and the fifth days of ICU admission were significantly shorter (*p* < 0.05) in the myocardial injury group than the non-myocardial injury group (64.17 ± 10.91 *vs* 101.36 ± 23.91, 67.07 ± 12.78 *vs* 100.07 ± 26.11, 70.63 ± 13.77 *vs* 106.84 ± 28.00, 74.94 ± 13.99 *vs* 100.49 ± 19.99 and 69.87 ± 12.23 *vs* 92.80 ± 23.21 respectively) (Table 2).

The mean (SD) RMSSD (ms) measured at the first, second, third, fourth, and the fifth days of ICU admission were significantly shorter (*p* < 0.05) in the myocardial injury group than the non-myocardial injury group (17.27 ± 2.83 *vs* 26.68 ± 5.33, 18.05 ± 2.89 *vs* 28.36 ± 5.42, 19.51 ± 3.36 *vs* 27.19 ± 5.63, 18.41 ± 2.69 *vs* 26.95 ± 5.39 and 20.62 ± 4.04 *vs* 28.12 ± 5.38 respectively) (Table 2).

## Discussion

In this retrospective cohort study, the data from 241 patients were examined of whom 54 were excluded or had incomplete data, the remaining patients were assigned to 42 in the myocardial injury group and 145 in the non-myocardial injury group. The main results of our study showed that the indices of HRV, including R-R intervals, SDNN and RMSSD, were greatly affected in COVID-19 patients who had myocardial injury.

This retrospective study was performed on the data derived from COVID-19 patients who were admitted to ICU and compared HRV indices between patients who developed myocardial injury and those without myocardial injury. Heart rate variability provides a clinically valuable and reliable quantitative measurement for alterations in the human body physiologic state [14]. In this study, we measured HRV using an ambulatory 5 min ECG recording from lead II. Both SDNN and RMSSD are indicators of gradual heart rate changes, therefore acting as sensitive and rapid indicators for sympathetic nerve function assessment [15]. This study demonstrated a strong correlation between both SDNN and RMSSD and CK, CKMB, Troponin levels and ST segment elevation in COVID-19 infected group. Added to that, RMSSD correlated positively with EF in the same group. In COVID-19 with the myocardial injury group, RMSSD correlated negatively with CKMB, Troponin levels and positively with the ejection fraction. Meanwhile, in the COVID-19 without myocardial injury group, only RMSSD correlated in a negative way with CKMB level.

Although literature about COVID-19 is relatively deficient; evidence of associated COVID-19 autonomic dysfunction is starting to grow. This is similar to autonomic dysfunction occurring in other viral infectious diseases such as human immunodeficiency virus, Epstein Barr virus, cytomegalovirus, dengue fever, tetanus and botulism [16]. The myocardial electrical stability is largely dependent on the equilibrium between vagal and sympathetic nerve activities. This equilibrium affects HRV, hence it can be used to evaluate the severity of ventricular arrhythmias [17]. Under physiological circumstances, vagal nerve is the principal nerve input at the sinoatrial node cells with secondary sympathetic input. Therefore, the effect of the vagal nerve dominates that of the sympathetic nerves [18].

Changes in autonomic nerve activity could be reflected by variations in the R-R intervals [19]. If vagal nerve activity increases, HRV will increase and protects against ventricular fibrillation, but if sympathetic nerve activity increases, HRV will decrease and might develop malignant arrhythmias. Rhythmic changes in HRV could also reflect changes in autonomic nerve function with high-sensitivity [20].

Our study verified low HRV in COVID-19 infected patients. This finding is consistent with other studies which reported a strong correlation between viral infection and autonomic dysfunction. Vijayabala et al. reported sympathetic dysfunction as one of the precipitated mechanisms for Dengue fever-associated shock syndrome [21]. Similarly, Griffin et al., in their studies, reported an abnormal HR pattern with decreased variability and transient deceleration, which proceed neonatal/infant sepsis [22, 23]. Carter et al. [24], had assessed HRV of 27 children during defervescence in dengue viral infection. He concluded that the cardiac parasympathetic activity was the major cause of reduced heart rate variability during this critical period of illness. He used the frequency domain to measure HRV after being corrected for baseline changes in heart rate [16]. In contrast to our study, Billman GE reported that low frequency/high frequency (LF/HF) data cannot accurately quantify cardiac “sympatho-vagal balance” either in health or disease. Particularly, the complex nature of LF power has a poor relationship to the sympathetic nerve activation, and the non-linear interactions between sympathetic and parasympathetic nerve activity [25]. La-Orkhun et al. [26], had investigated HRV as an indicator of autonomic function in patients with Dengue fever, and found insignificant changes in various time and frequency domain metrics of HRV at least 24 h after defervescence and follow-up 14 days after defervescence.

This study found lower HRV in COVID-19 patients who developed myocardial injury compared to COVID-19 patients without myocardial injury. COVID-19 is recognized as a potential cause of myocardial injury. The exact mechanism of COVID-19 induced cardiac injury remains unclear. This may include a direct myocardial involvement mediated by ACE2, cardiac myocyte apoptosis caused by cytokine storm and imbalanced response among subtypes of T helper cells, with hypoxia-induced excessive intracellular calcium load [27–29]. Eventually, infected cardiomyocytes would be lysed, with the development of left ventricular dysfunction [30].

Additionally, coronaviruses have been linked to myocarditis in patients of all age groups [31, 32] which is associated with ventricular arrhythmia [33, 34]. COVID-19 can cause coronary spasm, plaque rupture or micro-thrombi resulting from systemic inflammation or cytokine storm. This may explain the associated acute coronary syndrome [35, 36]. Added to that, heart failure is a commonly observed complication of COVID-19. It can be triggered by high fever, tachycardia and renal impairment [37, 38].

Increased levels of biomarker, e.g., elevated level of high-sensitivity cardiac Troponin T (hsTnT), reduced left ventricular ejection fraction, and increased native T1 and T2 (quantitative assessments of the myocardium composition), are usually indicators of myocardial injury with signs of inflammation [10].

In accordance with our study, Li et al. reported a reduction in HRV in patients with infective myocarditis because of the excessive enhancement of sympathetic activity and the inhibition of vagal nerve output [39]. Previous reports suggested that patients with chronic heart failure have reduced HRV with a significant correlation between the severity of left ventricular dysfunction and the extent of parasympathetic impairment [40]. Also, Casolo et al. reported that HRV evaluated during the acute phase of myocardial infarction is closely related to the severity of clinical and hemodynamic indices [8].

This study has some limitations: Firstly, we excluded any patients with associated co-morbidities that might have a perplexing effect on our observations, which made this study as a small-scale one. Also, this is a single hospital study and its results lack generalizability. Additionally, we could not find similar research on COVID-19 patients to compare our results with. We recommend similar prospective multi-center study with a large sample size, which might facilitate further evaluation of COVID-19 infection and autonomic dysfunction.

## Conclusions

Generally, COVID-19 infection is associated with lower heart rate variability in comparison with normal persons. Similarly, COVID-19 infected patients with myocardial injury showed a low degree of HRV compared with non-myocardial affected COVID-19 infected persons. The HRV analysis is correlated with other parameters used for myocardial injury diagnosis. Based on that it could be used as a bedside indicator which might alarm myocardial injury associated with COVID-19 infection.

## Data Availability

The datasets used and/or analyzed during the current study are available from the corresponding author on reasonable request.

## Abbreviations

HRV: Heart rate variability
ICU: Intensive Care Unit
SDANN: Standard deviation of NN intervals
RMSSD: The square of successive heartbeat interval differences
CHF: Congestive heart failure
HTN: Hypertension
HR: Heart rate
